# Exploring Physiological Linkage in Same-Sex Male Couples

**DOI:** 10.3389/fpsyg.2020.619255

**Published:** 2021-01-18

**Authors:** Xiaomin Li, Ashley Kuelz, Savannah Boyd, Kristin August, Charlotte Markey, Emily Butler

**Affiliations:** ^1^Department of Family Studies and Human Development, The University of Arizona, Tucson, AZ, United States; ^2^Department of Psychology, The University of Arizona, Tucson, AZ, United States; ^3^Department of Psychology, Rutgers University, Camden, NJ, United States

**Keywords:** physiological linkage, relationship functioning, *rties* package, same-sex male couples, conversational context

## Abstract

We explore physiological linkage (i.e., covariation of physiological channels between interacting partners; PL) among 34 same-sex male couples. Interbeat interval, an indicator of cardiovascular arousal, was collected across four conversational contexts in the lab: (1) a baseline period that did not involve conversation, (2) a conversation about body image, (3) a conversation about health goals, and (4) a recovery period that allowed for unstructured conversation. We used a newly developed R statistical package (i.e., *rties*; Butler and Barnard, 2019) that simplifies the use of dynamic models for investigating interpersonal emotional processes. We identified two different PL patterns: (1) a simple one that was characterized by stable synchronization and low frequency of oscillation; and (2) a complex one that was characterized by drifting synchronization, high frequency of oscillation, and eventual damping. Guided by social baseline theory and the reactive flexibility perspective, we explored the interactions between couple relationship functioning (i.e., love, conflict, commitment, sexual satisfaction, and relationship length) and conversational context as predictors of the PL patterns. The results suggest that partners in well-functioning relationships and emotionally challenging situations may be especially likely to show complex PL patterns that may reflect (or support) coregulatory processes.

## Introduction

Social relationships often provide health supporting benefits, but they can also be stressful if they involve conflict, threat of evaluation, or ambivalent emotions (Saxbe et al., 2020). Coregulation may be one mechanism determining whether a given relationship is helpful or harmful for the people involved. Coregulation refers to social partners becoming psychologically, behaviorally, and biologically intertwined in ways that support allostasis, which refers to stability through change, or the continual adjustment of multiple systems to maintain homeostatic balance (Sbarra and Hazan, 2008; Butler and Randall, 2013; Saxbe et al., 2020). Successful coregulation may help social partners negotiate any challenges that arise in their relationship, as well as achieve joint goals. In the biological domain, coregulation has been referred to as “physiological entanglement” or “physiological linkage” (Palumbo et al., 2017). Physiological linkage (PL) is indicated by the covariation of physiological channels between interacting partners and may provide a biological substrate for, or manifestation of, interpersonal coregulatory processes (Butler and Randall, 2013).

PL has been a focal area in the study of interpersonal relationships since the 1950s (e.g., Di Mascio et al., 1955) and offers several strengths for investigating interpersonal coregulatory processes. First, researchers can assess PL in second-by-second intervals, and such high time-resolution can reveal the nuances (e.g., fluctuations toward and away from stable emotional levels) in interpersonal dynamics and relationship functioning (Reed et al., 2013). Second, PL is unconscious and automatic, but may reflect partners’ emotional responding and efforts to influence each other. As such PL may provide a more sensitive measure than self-reports for interpersonal processes that are not readily accessible to awareness for many people (Butler and Randall, 2013). Third, associations have been found between PL and mental and physical health (Butler, 2017; Wilson et al., 2018), suggesting that if we had a better understanding of PLs, it may offer novel interpersonal interventions.

Despite the rapidly growing literature on PL, several important questions remain. To begin with, when operationalizing and quantifying PL, the majority of work has used simple indicators such as cross-correlations, which pick up bi-directional associations between partners’ physiology, but may fail to fully capture the complexity and diversity of PL patterns. For example, most common methods cannot distinguish the substantial differences between a PL pattern in which both partners’ physiological activity *dampens* together across time, which results in a stable homeostatic interpersonal biological state, and another PL pattern in which both partners’ physiological activity *amplifies* simultaneously across time, which produces an unstable or volatile interpersonal biological state (Butler and Randall, 2013; Reed et al., 2015). This methodological shortcoming may be the primary reason that PL has been widely associated with both desirable and undesirable variables, such as better health and higher relationship quality on the one hand, but stress and conflict on the other (Timmons et al., 2015; Palumbo et al., 2017; Saxbe et al., 2020).

Second, no studies we are aware of have examined PL in same-sex couple relationships (for similar arguments, see Timmons et al., 2015; Palumbo et al., 2017). Yet, the existing literature suggests one potential uniqueness of PL in same-sex couples. Specifically, in one study of heterosexual couples, the pattern when predicting men’s emotional experience from the female partners was different compared to that when predicting women’s emotional experience from male partners, with an in-phase pattern (e.g., partner’s emotions moving in the same direction) emerging for predicting men and an anti-phase pattern (e.g., partner’s emotions moving in the opposite direction) emerging for predicting women (Randall et al., 2013). Such gender differences in heterosexual couples may no longer exist in same-sex couples and may manifest as different patterns of PL.

To fill these gaps, we used secondary data from a larger project that focused on body image and health goals, as well as relational well-being, among same-sex male couples. We also used a newly developed R statistical package (i.e., *rties*; Butler and Barnard, 2019) that simplifies the use of dynamic models for investigating interpersonal processes, which enabled us to estimate complex patterns of PL. These data and analytic methods allowed us to address three research questions: (1) Would distinct patterns of PL emerge across experimental tasks varying in levels of interpersonal challenge? (2) Would distinct patterns of PL be associated with indicators of relationship quality? and (3) Would associations between PL patterns and relationship quality depend on the context (e.g., the experimental task)?

## Theory and Emprical Studies

### Introducing Physiological Linkage

Although physiology is typically viewed as an intrapersonal phenomenon, the physiology of two people can display substantial correlation (Levenson and Ruef, 1992). One basic distinction that needs to be made is between simple or stable PL and more complex or dynamic PL. For example, by sharing the same stimulus (e.g., watching a scary movie together), a simple in-phase PL pattern (e.g., partners’ physiologies change in the same direction) can automatically emerge as partner’s emotional responses covary in unison (Parkinson, 2011). A similar pattern may also arise in conversations involving low arousal emotions, such as collaborating on an interesting task or discussing the events of the day (Palumbo et al., 2017). Conversely, an anti-phase pattern of PL (partners’ physiologies change in opposite directions) may emerge when partners engage in trivial talk, possibly due to the nature of conversational turn-taking (Reed et al., 2013; Helm et al., 2014). In summary, fairly simple patterns – either in-phase or anti-phase – arise even in mundane situations and even between strangers (Palumbo et al., 2017). PL can become more complicated, however, when partners become emotional or attempt to regulate each other either consciously or automatically (Butler and Randall, 2013; Butler, 2017). For example, for some couples, the two partners’ physiologies can be changing in the opposite direction (i.e., anti-phase PL) and also amplify away from each other over time; for other couples, the two partners’ physiologies can switch from anti-phase to in-phase and then dampen together (Reed et al., 2015).

A large number of complicated PL patterns can be assessed by taking into consideration three characteristics of physiological signals, based on the assumption that PL takes the form of an oscillating pattern of fluctuations around a stable physiological basis (also called homeostasis or allostasis; Butler, 2011). The characteristics are: (a) frequency of oscillation (i.e., number of oscillations per unit of time), (b) damping and amplification (i.e., negative feedback loops that reduce arousal and stabilize the physiological signal across time, versus positive feedback loops that amplify physiological arousal away from homeostasis across time), and (c) coupling (i.e., whether two partners’ physiologies become coordinated or uncoordinated across time; Steele and Ferrer, 2011; Helm et al., 2012; Reed et al., 2015). Specific combinations of these three characteristics produce qualitatively and quantitatively different PL patterns. One pattern that has been noted in the literature involves anti-phase, damping oscillation; this pattern may indicate co-regulation, because the two partners are returning to homeostasis together across time. A second pattern that has been noted involves in-phase, amplifying oscillation; this pattern may indicate co-dysregulation, because the two partners increasingly deviate from homeostasis (Reed et al., 2015). Further variation can arise in the frequency of oscillation, suggesting that some couples can experience faster co-regulation/co-dysregulation than others (Helm et al., 2012).

In summary, PL can be understood as a multifaceted phenomenon in which frequency, damping/amplification, and coupling (or lack thereof) jointly give rise to complexity and diversity in the dynamic trajectories of two partners’ physiological signals. Yet, the lack of proper statistical tools has prohibited the exploration of such diverse PL patterns (see Helm et al., 2018). Therefore, to extend the existing literature, we relied on *rties*, a new R statistical package (Butler and Barnard, 2019), to model potentially complex PL patterns. We take a context-specific and couple-centered approach, meaning that we model the dynamics for each couple separately for each of the experimental tasks. We then investigate whether PL patterns vary across tasks, across couples, or across both.

### Associations Between Relationship Functioning and Physiological Linkage

#### Social Baseline Theory

Social baseline theory is one of the most widely applied theories in the field of relationships and health. It suggests that a relationship provides a context in which PL unfolds, and that the quality of the social relationship can promote or diminish PL patterns contributing to psychological and physical health (for similar arguments, see Sbarra and Hazan, 2008; Holt-Lunstad et al., 2010; Helm et al., 2012). In particular, when individuals are embedded in a predictable and familiar relationship, the security provided by the relationship can be used as an automatic, unintentional default strategy for maintaining a desirable emotional state (Beckes and Coan, 2011). Simply being around a secure partner, or even just thinking about them, reduces stress responding at both psychological and biological levels (Sbarra and Hazan, 2008; Holt-Lunstad et al., 2010). Moreover, as the levels of interdependence, shared goals, and joint attention in a relationship increases, the default strategy costs less effort and energy (Coan and Sbarra, 2015). Thus a couple’s relational context, in terms of habitual functioning and how much effort partners need to expend when interacting with each other, may be associated with different PL patterns.

#### Empirical Studies Based on Social Baseline Theory

In line with social baseline theory, some researchers have found associations between PL and variables connected to relationship functioning (e.g., relationship satisfaction, conflict, and the level of demanding or withdrawal behaviors; for reviews, see Butler, 2017; Palumbo et al., 2017). Yet, the results of these studies have been complex and ambiguous (Butler, 2017). Some studies suggest that high conflict and high withdrawing behaviors, presumably both indicators of distressed couple relationships, relate to in-phase PL (e.g., Reed et al., 2013; Gates et al., 2015). In contrast, other studies suggest that in-phase PL is particularly likely to occur when relationship satisfaction is high (Helm et al., 2014).

Such conflicting results may be partially due to the fact that existing studies focused on the associations between only one aspect of PL (the overall degree of covariation; e.g., Helm et al., 2014) and couple relationship functioning indices. The majority of prior studies have not considered that diverse patterns of PL are perhaps better understood by considering its multiple aspects (including frequency, damping/amplification, and coupling) as a totality (Gates and Liu, 2016). Thus in the present study we revisited the connection between couple relationship functioning and PL using statistical tools that allowed us to identify complex PL patterns based on constellations of multiple aspects of the oscillating physiological signals.

Given the exploratory nature of the present study, we decided to investigate associations between multiple aspects of relationship functioning (i.e., love, conflict, sexual satisfaction, and commitment) and PL patterns. An examination of these variables allow us to relate our results to existing studies, which used similar constructs [i.e., the feeling of love and intimacy in Helm et al. (2014); conflict in Koole and Tschacher (2016); sexual satisfaction in Freihart and Meston (2019); the feeling of being committed in Helm et al. (2014)]. We also included relationship length as another potential predictor for PL patterns, primarily given that longer relationship length indicates higher interdependence between spouses (Campbell et al., 2006; Knight, 2011).

### The Moderating Role of Conversational Context

#### Reactive Flexibility Perspective

Another factor that may have contributed to ambiguous findings regarding associations between couple relationship functioning and PL is the moderating role of context (i.e., conversational contexts). More specifically, PL patterns may vary as two partners negotiate the demands and goals of different types of conversation (e.g., cooperating on a topic, resolving a conflict, etc.) and adjust their efforts to influence each other accordingly (i.e., the reactive flexibility perspective; Hollenstein, 2015; Butler, 2017). For example, on the one hand, PL patterns may be as simple as basic anti-phase turn-taking in casual conversations or low-level in-phase synchrony when discussing a mildly interesting topic. On the other hand, however, they may be as complicated as anti-phase-to-in-phase transitions with amplification in a highly competitive conversation (Helm et al., 2014; Reed et al., 2015).

#### Emprical Studies Supporting Reactive Flexibility Perspective

In line with this idea, researchers have consistently found interactive effects between couple relationship functioning indices and conversational contexts in connection with PL (for a review, see Palumbo et al., 2017). Thus, in the present study we investigated a series of conversational contexts that might induce different motivations to influence the partner, and explored whether the associations between multiple aspects of couple relationship functioning (e.g., love, conflict, etc. listed above) and qualitatively distinct patterns of PL varied across contexts.

### Body Image and Health Goal Conversations Among Same-Sex Male Couples

In the current study with same-sex male couples, we focused on body image and health goal conversations. Generally, body image and health goals are serious relational topics that partners are likely to be motived to engage in (either with collaboration or argument; Smith et al., 2009; Burke et al., 2012). Such conversations may be even more salient and arousing in same-sex male dyads. In particular, and in comparison to their heterosexual counterprts, some evidence suggests that gay men hold more unrealistic thoughts about body ideals and are more concerned with gaining weight (McClain and Peebles, 2016; Brewster et al., 2017). Similarly, partners in same-sex male couples may be particularly unsatisfied with each other’s body and weight, which results in especially high levels of intention and motivation to exert influence on the other’s feelings and health behaviors (Theiss et al., 2016). Therefore, we systematically varied conversational context by asking the couples to engage in: (1) a baseline context in which no conversation took place, (2) loosely structured conversations about body-image, or (3) health-goals, and finally (4) free unstructured conversations.

### Exploratory Hypotheses

Given the exploratory nature of our study and lack of definitive prior literature, we did not specify detailed hypotheses, but instead used cross-validation to avoid over-fitting the data and to increase the chances that the results would replicate in a new sample (see “Analytic Approach” for details). In general, however, based on the literature reviewed above we expected: (1) at least 2 distinct PL patterns would emerge, with a simple pattern occurring most often in the non-challenging baseline and unstructured conversations and a more complex pattern emerging during the body image and health goal contexts, given that they would presumably elicit more emotion and attempts at regulation, and (2) more complex PL would be associated with higher relationship quality, especially during the challenging conversations (body image and health goals), because although negative emotions may be aroused by those contexts, partners in a secure relationship may be more effective at regulating each other’s emotion and behaviors, such that their initial coupled stress responses eventually return to homeostasis.

## Materials and Methods

### Participants

Data in the present study come from a larger project that examined associations between romantic relationships and health among male same-sex romantic couples who had been together for at least 6 months (for a detailed description, see Markey et al., 2014). The present study included a sub-sample of 34 couples from whom usable physiological data was collected. Although the final sample is somewhat small, based on a systematic review, it was comparable to the median of sample size in existing studies that examined physiological linkage in romantic relationships (Palumbo et al., 2017). Further, we used Bayesian analysis which is more reliable with small samples (see below) and our goals were exploratory, not confirmatory, which also mitigates concerns about the modest sample. For the 34 couples included in the present study, participants varied in age (Range = 19–71 years, *Mean* = 31 years), race/ethnicity (69.1% non-Hispanic White, 30.9% minority group), household income (Range = under 20K to 100K or more, *Median* = 50–70K), and relationship length (Range = 0.5–34 years, *Mean* = 6.3 years).

### Procedures

The research procedures were approved by the IRB at the institution where the research was conducted. Couples were recruited via advertisements in a variety of periodicals and at health and wellness centers, as well as LGBTQ + centers in the Philadelphia metro area. After being screened for eligibility via phone or a web-based survey, couples visited the researchers’ laboratory to complete the study. To ensure privacy, the two partners in a couple were placed in separate rooms to complete the first part of the survey (i.e., survey related to body image, weight management behaviors, relationship with their partner, etc.).

Then, participants were asked to sit at a table in a small, distraction-free room to engage in the following social situations. Each situation lasted about 10 min. In the baseline situation, partners started working on the second part of the survey (survey about background information, personality, support received, etc.), during which little or no conversation took place. After the baseline, participants engaged in two conversations that were arranged in a counterbalanced sequence: a body image conversation and a health goal conversation. During the body image conversation, participants were asked to talk about what they thought about their own and their partners’ body size and weight issues. In the health goal conversation, participants first listed their own health goals, next discussed and agreed on three shared goals that worked for themselves and their partners, and then figured out how to work together with their partners to accomplish the three shared goals. After completing the body image and health goal converations, participants had a recovery period when they could finish the second part of the survey (if needed) and talk freely with their partners. Each couple was compensated $100 for the time.

### Measures

#### InterBeat Interval

Interbeat interval (IBI) refers to the time in milliseconds between subsequent R waves (the peaks in an electrocardiogram signal) and is an indicator of fluctuations in heart rate. In general terms, IBI is an index of arousal, regardless of the source of the arousal. In other words, IBI fluctuations are not indicative of valence (e.g., positive vs. negative), only of activation. One advantage of IBI for our purposes is that it is very dynamic, meaning that it changes over a time range of a few seconds, allowing us to assess between-partner PL with fine-grained temporal precision. In contrast, other measures of autonomic physiology, such as electrodermal activity (EDA) or heart rate variability (HRV), are slower moving. Another advantage of IBI for our purposes is that it is controlled by both the sympathetic and parasympathetic systems, acting in coordination with each other. As such it reflects the full range and complexity of autonomic activity, making it sensitive to both activating influences (via the sympathetic system) and damping or de-activating influences (via the parasympathetic system). In contrast, EDA and HRV are driven uniquely by the sympathetic and parasympathetic systems respectively, making them more specific, but less likely to pick up the full range of PL.

IBI was measured by electrocardiogram (ECG) for all participants continuously throughout the interaction. ECG was recorded with electrodes in the modified Lead II placement and sent to a computer via Biopac ECG100C Module and MP150 amplifier (Biopac Systems, Inc., Goleta, CA). To extract the interbeat interval (i.e., IBI;), the ECG data were scored with Acknowledge version 4.4 (Biopac Systems, Inc., Goleta, California) and aggregated in 10-second units.

#### Love

We used the 10-item love subscale from the Marital Interaction Scale (MIS; Braiker and Kelly, 1979). One example item is “How close do you feel toward your partner?” On each item, participants indicated the extent to which the statement described their feelings about their romantic partner. Responses ranged from 1 (Not at all) to 9 (Very much). Items were summed to create a scale score, and higher scores indicate higher love. Cronbach’s α was 0.76.

#### Conflict

We used the 5-item conflict subscale from the Marital Interaction Scale (MIS; Braiker and Kelly, 1979). One example item was “How often do you and your partner argue with one another?” On each item, participants indicated the extent to which the statement described their feelings about their romantic partner. Responses ranged from 1 (Not at all) to 9 (Very much). Items on were summed to create a scale score, and higher scores indicated higher conflict. Cronbach’s α was 0.69.

#### Sexual Satisfaction

We used the 25-item, unidimensional Index of Sexual Satisfaction scale (Pepe and Byrne, 1991). An example item was “sex with my partner has become a chore (reverse scored).” On each item, participants indicated the extent to which they agreed with the statement. Responses ranged from 1 (Strongly disagree) to 5 (Strongly agree). With reversed items recoded, items were averaged to calculate the scale score, and higher scores indicated higher sexual satisfaction. Cronbach’s α was 0.91.

#### Commitment

We used the Multiple Determinants of Relationship Commitment Inventory, which included 30 items for 6 dimensions: rewards, match to ideal comparison level, investments, barriers, costs, and alternatives (Kurdek, 1995). Responses ranged from 1 (Disagree strongly) to 5 (Agree strongly). We first reversed scored items for costs and alternatives and then averaged all 30 items to calculate the sum score of commitment, with higher scores indicating higher commitment. Cronbach’s α was 0.85.

#### Relationship Length

One open-ended question was used to measure relationship length: “For how many months have you been continuously romantically involved with your partner?”

### Analytic Approach

We conducted analyses using the R Statistical Platform, version 3.6.3 (R Core Team, 2020). Analyses proceeded in the following two stages:

#### Stage 1: Modeling Physiological Linkage

In the present study, couples completed 133 conversational contexts in total (34 couples × 4 contexts each, with three couples having missing data for 1 of the contexts). For each context completed by each couple, we used the *rties* package version 5.0.0 (Butler and Barnard, 2019) to estimate a Coupled Oscillator (CO) model of IBI linkage over time. The vignettes that accompany the *rties* package provide extensive documentation of the approach. In brief, the CO model in *rties* takes the form of a regression model predicting the second-derivative of the observed variable (in this study, IBI for each partner) from 8 predictors: (a) each partner’s own IBI time series (related to the frequency of oscillations), (b) the first derivative of each partner’s own IBI time series (related to damping/amplification, (c) each person’s partner’s IBI time series (coupling with respect to frequency), and (d) each person’s partner’s first derivative of their IBI time series (coupling with respect to damping/amplification). The *rties* package uses an idiographic approach and applies the regression model to each context completed by each dyad, one context at a time. As such, eight regression parameters were generated (i.e., four for each partner) based on IBI collected from each couple in each context. Across all conversational contexts completed by all couples, the average number of valid IBI data was 120 (i.e., *n* = 120 when estimating eight regression parameters).

The CO model requires individual-level, distinguishable data from the two partners (e.g., there must be some way to distinguish what data came from which partner), but in the present study the partners are indistinguishable, due to being same-sex and not otherwise systematically different from each other. To address this, we created an arbitrary distinguishing variable (“A vs. B”), such that in each couple one partner was randomly assigned as “A” and the other as “B”. This allows estimation of the CO model (which would not change if the random assignment was reversed for some or all couples), but no meaningful interpretation of the distinguishing variable is generated (see further explanation in the caption for Table 2).

Data for a CO model should first be linearly detrended (Boker and Laurenceau, 2006) and the *rties* package provides the tools to do so. Next, the first and second derivatives of the observed variable need to be estimated from the data (i.e., using a Local Linear Approximation; Boker and Nesselroade, 2002). This approach has notable limitations, but is tractable with relatively little knowledge about linear dynamic systems and is the approach implemented by *rties* (Butler and Barnard, 2019). To do so, users need to specify 3 parameters: delta, tau, and embed. Delta refers to the inter-observation interval, tau is the number of time points to include when estimating the first derivative, and embed is relevant to the degree of derivatives that are desired. As we needed to estimate the second derivative, the minimum embed was 3. In the present study, we set delta to 1, so that every observation was utilized for fitting. The vector of tau included 1 and 2. The vector of embed included 3 as the sole element. The *rties* package fits a CO model to each dyad’s data for each context multiple times using all combinations of the embed and tau values and returns the combination that maximized the *R*^2^ for each couple in each context. This *R*^2^ information can be used to determine how well the model fits the data both for each couple and on average across couples. The estimated period of oscillation is also returned and the 8 parameters for each couple in each context (described earlier) are stored as a new data frame.

Lastly, *rties* allows the user to include the set of 8 parameter estimates for each couple in each context as indicators for a Latent Profile Analysis (LPA), to derive qualitatively distinct groups of all couple-context combinations based on their dynamic linkage patterns (*n* = 133 for LPA in this study, as we have 133 conversational tasks completed by 34 couples). This approach is taken because the CO model assesses non-linear dynamics across time, which means the behavior of the dyadic system cannot be understood by interpreting individual parameters in isolation, as is possible with a linear model. The LPA allows the CO parameters to act together (versus in isolation) to estimate qualitatively distinct groups of dyads that reflect the potentially complex, dynamic trajectories for both partners in each context. The prototypical trajectories for each profile can then be plotted based on the profile’s average values of the 8 parameters.

#### Stage 2: Predicting Physiological Linkage

The purpose of stage 2 is to predict physiological linkage patterns identified in the LPA for each couple in each context (based on the profile groupings identified in stage 1) from relationship traits and the four conversation contexts. Given the non-independence among the four contexts experienced by each couple, we conducted Bayesian multilevel modeling (MLM) with each dyad allowed to have their own intercept. Estimation of MLM was conducted via *brms* 2.11.5, an R package that uses Stan to estimate Bayesian multilevel models (Bürkner, 2017, 2018). We preferred Bayesian to traditional Null-Hypothesis Significance Testing (NHST) for the following reasons: First, Bayesian analyses are less sensitive than NHST to sample size and will, therefore, generate more robust estimation for small-to-modest sized sample (as is the case with the present study in which *n* = 133 at Level 1 and 34 at Level 2; Branch, 2014). Second, Bayesian estimation reflects the uncertainty of the population parameter more accurately than NHST. In particular, NHST represents the uncertainty of the parameter using a confidence interval (CI), which reflects the upper and lower limits of values that may not be rejected by *p* < 0.05 but provides no probability estimate that the specific parameter value is within the range. In contrast, Bayesian estimation explicitly indicates the uncertainty of parameters by generating the posterior distribution (i.e., highest density interval (HDI); Kruschke and Liddell, 2018), which reflects the probability that the specific parameter is within the range, given the data and the model. As a result, Bayesian analysis allows researchers to make specific probability statements about each parameter.

We tested five sets of models (details are shown in Supplementary Table 1 of the Supplementary Material). As seen in the measures section, relationship length is a couple-level variable; love, conflict, sexual satisfaction, and commitment are individual-level variables. In each set of models, we tested the main effect of conversational context, the main effect of the couple relationship indices, and the interaction between conversational context and couple relationship indices. For models including individual-level variables, we considered both between-dyad variation (i.e., the average of the two spouses’ reports) and within-dyad variation (i.e., the discrepancy between the two spouses’ reports; an average-difference model; Kenny, 1996). This is a parsimonious strategy to fully account for reports of both spouses when exploring the associations between couple relationship and PL patterns among indistinguishable dyads. To test the potential moderating role of conversational context in models including individual-level variables, two interactive terms were generated and included: (1) the average between two spouses’ reports × context, and (2) the difference between two spouses’ reports × context. If either of these two interactive terms was not notable (e.g., the 95% HDI included zero), we then removed it to generate a simplified model.

Given the lack of relevant literature, we used the *brms* default, uninformative priors (see https://cran.rproject.org/web/packages/brms/vignettes/brms_multilevel.pdf for more details about the default prior distribution). For the final models we used 10 chains to generate posterior distributions (for each chain, number of iterations = 10,000, and burnin iterations = 5,000). We checked model convergence based on Rhats, effective sample sizes, and visualization of trace plots. All models in the Supplementary Document showed evidence of convergence, as well as stable results when fitting the model multiple times.

Next, we used cross-validation to compare all the models in each set to select the optimal one. Cross-validation (CV) is the gold standard for model comparison because it balances achieving a good fit for existing data, while avoiding over-fitting and hence improving generalization to future unseen data. Most standard model fit statistics, such as AIC and BIC were developed as approximations for cross-validation, but do not perform as well. We used leave-one-out (LOO) cross-validation given our relatively small sample size. This method of CV involves leaving out one data point at a time and building the model on the rest of the data. The model is then tested against the data point that was left out and the testing error is recorded. The process is then repeated for all data points and the overall prediction error is computed by taking the average of all test error estimates. Finally, the models (ranging in complexity) are compared and the best fitting model is chosen based on the expected log predictive density (ELPD) difference, relative to its standard error (SE). The smallest ELPD indicates the model that best fits the unseen data, and an ELPD difference between two models that is smaller than 2 SEs indicates equivalently fitting models for unseen data (Vehtari et al., 2017).

Finally, to further protect against Type-I errors, we considered Regions of Practical Equivalence (ROPEs) when deciding between equivalently fitting models. ROPEs are a Bayesian technique that establishes a probability region around zero for a given parameter representing a chosen effect size. We used an effect size of ±0.1, which is the standard range for representing an effect so small that we might as well treat it as zero for practical purposes (Kruschke, 2018). We then only consider parameters with a low probability of being in the ROPE range as credible results.

## Results

### Descriptive Analyses for Variables Connected to Relationship Functioning

Table 1 displays descriptive statistics for variables connected to relationship functioning. As can be seen, relatively large variability existed for individuals’ reports, the average between spouses’ reports in each couple, and the difference between spouses’ reports in each couple.

**TABLE 1:** Summary of relationship variables for 68 partners in 34 couples.

	**Mean**	**SD**	**Min**	**Max**
Love for each partner	75.5	8.43	50.00	90.00
Conflict for each partner	24.5	6.14	9.00	36.00
Sexual Satisfaction for each partner	3.60	0.72	1.75	4.92
Commitment for each partner	3.85	2.50	4.73	0.45
Average love for each couple	77.48	6.97	58.50	88.00
Difference in love for each couple (absolute value)	7.88	5.58	0.00	22.00
Average conflict for each couple	21.48	4.84	10.00	29.50
Difference in conflict for each couple (absolute value)	6.53	3.84	0.00	18.00
Average sexual satisfaction for each couple	3.84	0.62	2.08	4.80
Difference in sexual satisfaction for each couple (absolute value)	0.55	0.46	0.08	1.96
Average commitment for each couple	3.86	2.73	4.52	0.36
Difference in commitment for each couple (absolute value)	0.40	0.00	1.23	0.32
Relationship length in years	6.33	8.35	0.50	34.00

*For each relationship variable, we list estimates for the individual reports for all 68 partners; we also list the between-dyad variation (i.e., the average of the two spouses’ reports) and within-dyad variation (i.e., the difference between the two spouses’ reports; an average-difference model; Kenny, 1996) for all 34 couples.*

### Physiological Linkage Profiles for IBI

Table 2 displays descriptive statistics averaged over all contexts and couples for the 8 parameters estimated in the CO model, including the adjusted overall *R*^2^ value and the period of oscillation. With adjusted *R*^2^ ranging from.43 to 0.79, the CO model fit the data fairly well for all context/couple combinations. There was also relatively large variation across contexts and couples for all of the parameters, as well as the estimated period of oscillation. Given that we assessed IBI in 10-second units, the length of the average period was about 1.45 min for both partners (e.g., 8.7 units ^∗^ 10 s = 87 s/60 s = 1.45 min). Given that the average length of the conversation contexts was 10 min, about 7 cycles were included in each context, which is a reasonable number of cycles for assessing IBI dynamics.

**TABLE 2:** Summary of CO model parameters across the 133 contexts completed by 34 couples.

	**Mean**	**SD**	**Min**	**Max**
Frequency of oscillations (A)	–0.69	0.59	–2.83	–0.25
Damping/amplification (A)	0.01	0.22	–0.82	1.47
Coupling with partner in frequency (A)	0.00	0.20	–1.04	0.98
Coupling with partner in damping/amplification (A)	0.01	0.24	–0.46	1.55
Frequency of oscillations (B)	–0.65	0.47	–2.64	–0.24
Damping/amplification (B)	–0.02	0.11	–0.46	0.39
Coupling with partner in frequency (B)	0.01	0.16	–0.55	0.93
Coupling with partner in dampingen/amplification (B)	0.00	0.22	–1.28	0.63
*R*^2^	0.64	0.08	0.43	0.79
Period (A)	8.65	1.96	3.73	12.45
Period (B)	8.66	1.78	3.86	12.87

The distinguishing variable A/B was randomly assigned as these were indistinguishable dyads. Since the model was fit for each context within each couple, the parameters are never averaged across people and so keeping A and B separate is legitimate, despite the random assignment. In other words, the “A” and “B” distinguisher serves only to keep the two partners’ data within a context and a couple separate and reversing the order of who is “A” or “B” would simply switch the estimates for the “A” and “B” parameters for that context/couple combination. This is in contrast to a multilevel model, where the estimates are averaged over all “A” partners to get the “A” estimates and all “B” partners for the “B” estimates. In that case, the results could change substantially if the distinguisher were reversed for some couples. For period, the mean indicated the number of time units. As we assessed IBI in 10-second units, the length of the average period was about 1.45 min for both partners (e.g., 8.7 units * 10 s = 87 s/60 s = 1.45 min).

Based on the LPA with 8 CO parameters, we generated three solutions with 2, 3, and 4 profiles respectively. The 2-profile solution was chosen as optimal because: (a) the predicted IBI trajectories for the 2-profile solution were visually distinct, whereas the trajectories in 3- and 4- profile solutions had visually similar temporal patterns; and (b) the smallest profile in the 3- and 4- profile solutions did not include enough context/couple combinations (i.e., they included less than 10% of 133 contexts) and hence interpretation of the dynamics within these profiles was unlikely to be robust due to the solution being driven by a very small portion of the data.

**FIGURE 1:**
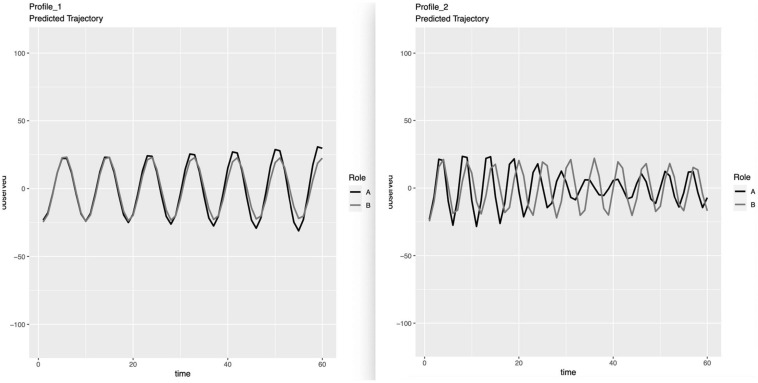
Estimated IBI trajectories for the two profiles. The first profile characterized 109 topic-couple combinations, while the second characterized 24. For indistinguishable dyads, the distinguishing variable A/B was randomly assigned and should not be interpreted. Predicted trajectories for Profile 1: The Simple Profile. Predicted trajectories for Profile 2: The Complex Profile.

Table 3 displays the average parameter estimates in Profile 1 (i.e., 109 of 133 context-couple combinations; 82.0%) and Profile 2 (i.e., 24 of 133 context-couple combinations; 18.0%). Some notable differences were observed in the frequency of oscillations, period, and coupling for damping/amplification. To better interpret the results of the 2-profile solution, we then plotted the dynamic trajectories predicted for each profile over the average length of contexts (i.e., about 10 min). As seen in Figure 1, Profile 1 was characterized by a relatively simple and stable temporal dyadic trajectory (i.e., stable in-phase synchronization, with lower-frequency of oscillation in comparison to Profile 2 and little amplification or damping). Thus, we labeled Profile 1 as the “Simple” profile. In contrast, Profile 2 was characterized as a higher-frequency oscillating process, with drifting synchronization (i.e., first in-phase, then anti-phase, and finally in-phase) and some evidence of damping over time. Thus, we labeled Profile 2 as the “Complex” profile.

**TABLE 3:** Summary of CO model parameters for the simple profile (109 context-couple combinations) and the complex profile (24 context-couple combinations).

	**Simple profile**		**Complex profile**	
		
	**Mean**	**SD**	**Mean**	**SD**
Frequency of oscillations (A)	–0.60	0.49	–1.11	0.83
Damping/amplification (A)	0.02	0.13	–0.03	0.45
Coupling with partner in frequency (A)	0.00	0.18	–0.01	0.27
Coupling with partner in damping/amplification (A)	–0.03	0.15	0.19	0.44
Frequency of oscillations (B)	–0.55	0.32	–1.08	0.75
Damping/amplification (B)	–0.01	0.09	–0.06	0.19
Coupling with partner in frequency (B)	0.00	0.13	0.01	0.26
Coupling with partner in dampingen/amplification (B)	0.01	0.22	–0.02	0.23
*R*^2^	0.64	0.08	0.65	0.08
Period (A)	8.91	1.62	7.47	2.84
Period (B)	8.99	1.48	7.14	2.25

Similar to the note in Table 3, A/B was randomly assigned since these were indistinguishable dyads. Switching some dyad assignments would simply change which trajectory was labeled “A” and which was labeled “B” for those dyads, without changing the overall pattern of the dynamic trajectories. For period, the mean indicated the number of time units. As we assessed IBI in 10-second units, the length of the average period was about 1.48 min for both partners (e.g., 8.9 units * 10 s = 89 s/60 s = 1.48 min) in the simple profile. The length of the average period was about 1.23 min for both partners (e.g., 7.4 units * 10 s = 74 s/60 s = 1.23 min) in the complex profile.

### Associations Between Linkage Profiles, Relationship Functioning, and Conversational Contexts

Table 4 shows the specifications for the final models chosen based on cross-validation. Full results for the cross-validation are provided in the Supplementary Table 2. In brief, within the set of models for a given predictor (e.g., love, conflict, etc.) we chose the model that either: (1) had the smallest ELPD (this applied to choosing the models for love, conflict and relationship length), or (2) had an ELPD that was less than 2 standard errors worse than the smallest ELPD and had credible effects larger than a 0.1 effect size for at least one of the additional predictors (this applied to choosing models for sexual satisfaction and commitment). These decision criteria ensure that all reported effects show some evidence of being larger than 0.1 in size and the models chosen were the optimal ones for predicting the unseen data.

**TABLE 4:** Specification of fixed effects for final models for each predictor variable. All models included a random couple intercept to account for nesting of contexts in couples.

**Predictor**	**Final model**
Love	Profile _contexti, dyad j_ = π_0j_ + π_20_ (Average love) + π_30_ (Partner difference in love)
Conflict	Profile _contexti, dyad j_ = π_0j_ + π_20_ (Average conflict) + π_30_ (Partner difference in conflict)
Sexual satisfaction	Profile _contexti, dyad j_ = π_0j_ + π_10_ (Context) + π_20_ (Average sex satisfaction) + π_30_ (Partner difference in sex satisfaction) + π_40_ (Context) × (Average sex satisfaction)
Commitment	Profile _contexti, dyad j_ = π_0j_ + π_10_ (Context) + π_20_ (Average commitment) + π_30_ (Partner difference in commitment) + π_40_ (Context) × (Average commitment)
Relationship length	Profile _contexti, dyad j_ = π_0j_ + π_10_ (Context) + π_20_ (Relationship length)

#### Love

**FIGURE 2:**
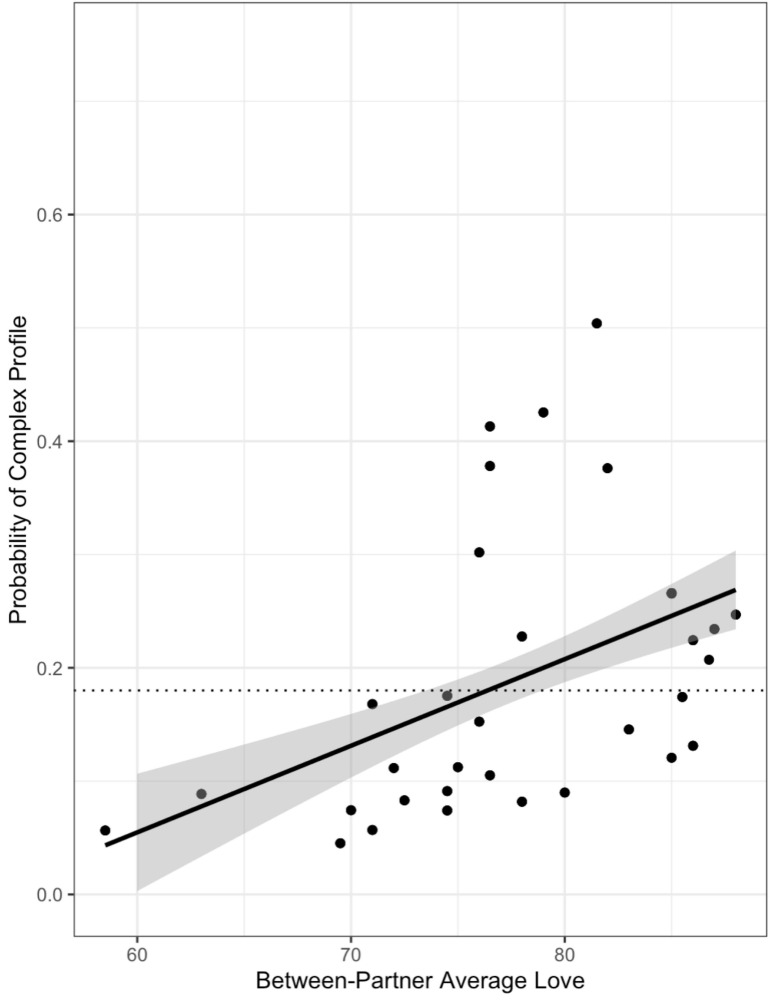
The main effect of average love across contexts. The dotted line shows the unconditional probability of being in the complex profile as a reference point. Higher average love was associated with a higher probability of being in the complex profile across contexts.

The final model for love included the main effects of the between-partner averages and differences in love. The *R*^2^ for the model was.18, which means that the model accounted for 18% of the variance in the profile probabilities. Results provided modest evidence that the main effect of average love was positive and non-zero. The posterior mean for the parameter was 0.11 and although the 95% HDI included zero, the 90% HDI did not (0.01–0.22). In addition, there was only a 19% probability that the effect was small enough to be in the ROPE. As shown in Figure 2, higher between-partner average love was associated with a higher probability of being in the complex profile regardless of context.

#### Conflict

**FIGURE 3:**
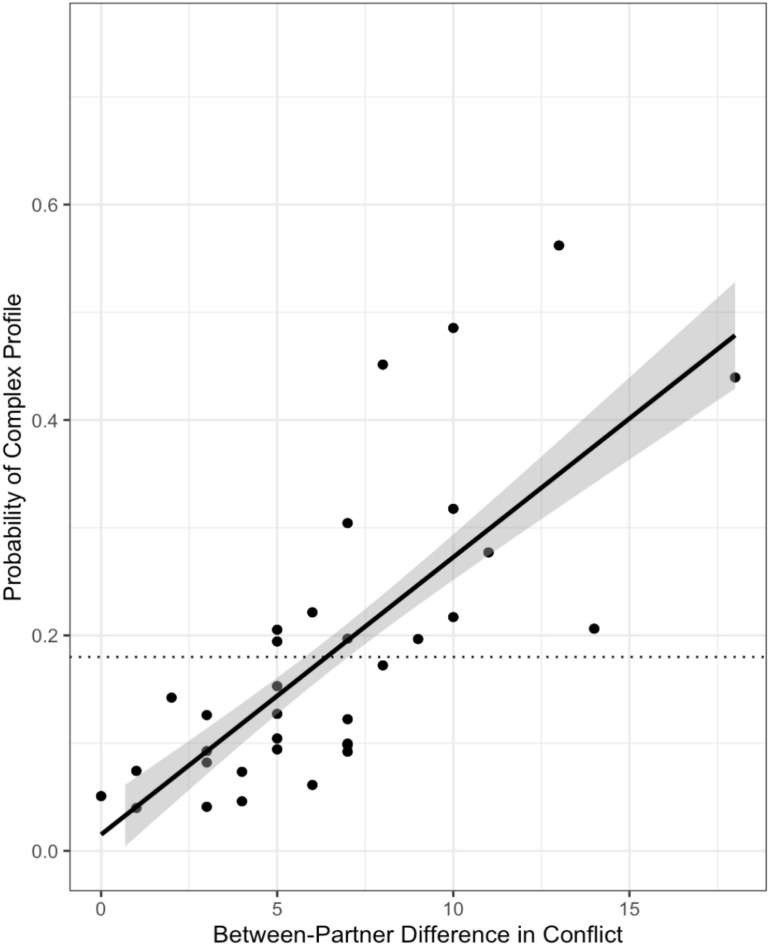
The main effect of between-partner differences in conflict across contexts. The dotted line shows the unconditional probability of being in the complex profile as a reference point. Larger between-partner differences in conflict were associated with a higher probability of being in the complex profile across contexts.

The final model for conflict included the main effects of the between-partner averages and differences in conflict. The R^2^ for the model was.19, which means that the model accounted for 19% of the variance in the profile probabilities. Results provided strong evidence that the main effect of the between-partner difference in conflict was non-zero, with the posterior mean for the parameter being 0.25 and the 95% HDI ranging from 0.08 to 0.46. In addition, there was zero probability that the effect was small enough to be in the ROPE. As shown in Figure 3, larger between-partner differences in conflict were associated with a higher probability of being in the complex profile regardless of context.

#### Sexual Satisfaction

**FIGURE 4:**
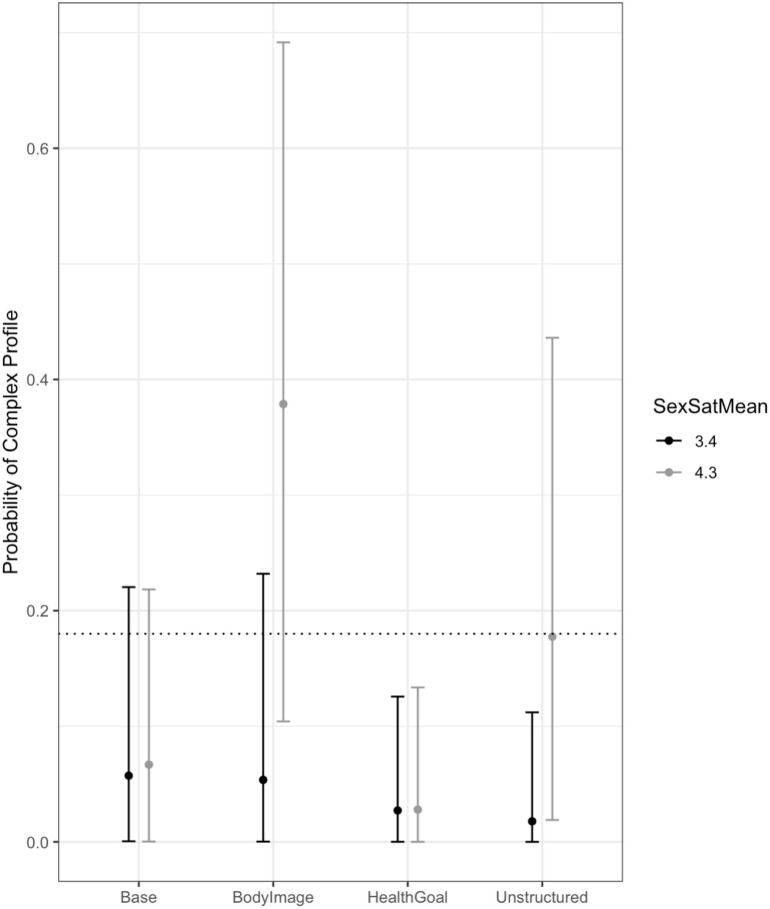
The interaction of average sexual satisfaction and context. The dotted line shows the unconditional probability of being in the complex profile as a reference point. Higher average sexual satisfaction was associated with a higher probability of being in the complex profile during the Body Image conversation.

The final model for sexual satisfaction included the main effects of the between-partner averages and differences in sexual satisfaction, along with the interaction of average sexual satisfaction and context. The *R*^2^ for the model was 0.34, which means that the model accounted for 34% of the variance in the profile probabilities. Results provided modest evidence that the interaction of average sexual satisfaction and context during the Body Image conversation was non-zero. The posterior mean for the parameter was 2.52 and although the 95% HDI included zero, the 85% HDI did not (0.13–4.63). In addition, there was only a 0.5% probability that the interaction between sexual satisfaction and context was small enough to be in the ROPE for the Body Image conversation. As shown in Figure 4, during the Body Image conversation, higher average sexual satisfaction was associated with a higher probability of being in the complex profile.

#### Commitment

**FIGURE 5:**
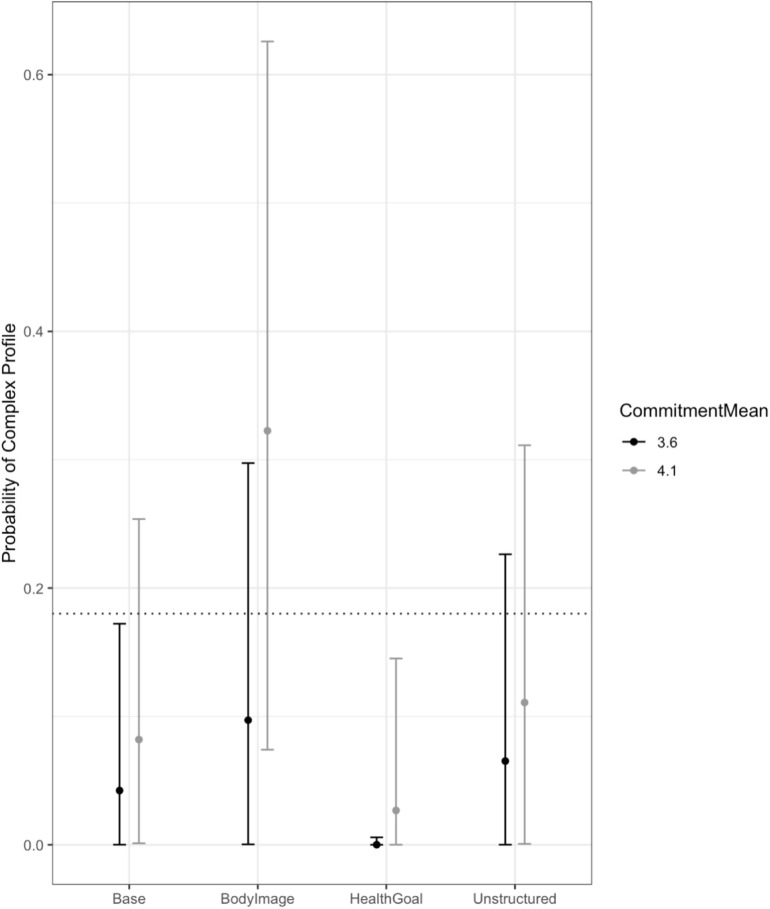
The interaction of average commitment and context. The dotted line shows the unconditional probability of being in the complex profile as a reference point. Higher average commitment was associated with a higher probability of being in the complex profile during the Health Goals conversation, although this is due to low commitment couples having an essentially zero probability of being in the complex profile while discussing health goals, rather than high commitment couples being likely to be in the complex profile during that conversation.

The final model for commitment included the main effects of the between-partner averages and differences in commitment, along with the interaction of average commitment and context. The *R*^2^ for the model was 0.35, which means that the model accounted for 35% of the variance in the profile probabilities. Results provided strong evidence that the interaction between average commitment and context was non-zero during the Health Goals conversation, with the posterior mean for the parameter being 12.35 and the 95% HDI ranging from 2.28 to 25.60. In addition, there was zero probability that the interaction between commitment and context was small enough to be in the ROPE for the Health Goals conversation. As shown in Figure 5, during the Health Goals conversation, higher average commitment was associated with a higher probability of being in the complex profile, although as can be seen in the figure, this is due to an essentially zero probability of low commitment couples being in the complex profile, rather than high commitment couples having a high probability of being in the complex profile.

#### Relationship Length

The final model for relationship length included the main effects of relationship length (in months) and context. The *R*^2^ for the model was 0.26, which means that the model accounted for 26% of the variance in the profile probabilities. Results suggested that there were no credible associations between either relationship length or context with the probability of being in the complex profile.

## Discussion

Social baseline theory argues that our neural processing has evolved to automatically assume that we are embedded in a supportive social network (Coan et al., 2006; Coan and Sbarra, 2015). In other words, our brain assumes social connection as the default situation and our homeostatic state is defined by interconnection with other people at all levels (e.g., psychological, behavioral, biological; Saxbe et al., 2020). Coregulation refers to the interpersonal processes that enable us to return to our baseline, e.g., our secure interpersonal state, when we are perturbed away from it (Sbarra and Hazan, 2008). Coregulation is a dynamic process, involving complex positive and negative feedback loops within and between people, across psychological, behavioral and biological channels (Butler and Randall, 2013). As such, it enables us to respond efficiently as an interpersonal system to challenges and opportunities, and then return to our interpersonal homeostatic baseline afterward. Social baseline theory further suggests that high quality relationships automatically reduce threat responding, thereby freeing up resources for social partners to engage flexibly with each other and the world (Coan et al., 2006; Coan and Sbarra, 2015). In other words, high quality relationships both promote and rely on coregulation.

Our exploratory study focused on the biological channel of coregulation, e.g., physiological linkage (PL) of interbeat interval (IBI), and extends the existing literature in the following ways: First, using a newly developed R statistical package (*rties*, Butler and Barnard, 2019), we explored diverse patterns of PL during both neutral social contexts and emotionally arousing ones (i.e., body image and health goal conversations) in an understudied population (i.e., same-sex male couples). Second, guided by the perspective of social baseline theory, we explored whether or not (and if yes, which aspect of) couple relationship functioning was associated with PL patterns in same-sex male relationships. Third, we explored the potential moderating role of conversational contexts in the associations between couple relationship functioning and PL patterns.

Given the lack of prior work distinguishing among different PL patterns, our research is exploratory and hence our expectations for what we would find were tentative. Nevertheless, we expected that we would find at least two distinct patterns, with one being some form of relatively simple synchrony and the other being more complex. Second, we expected that higher quality relationships would promote more elaborate forms of coregulation and would therefore be associated with more complex PL. Finally, we expected the more challenging conversation contexts (body image and health goals) would produce more complex PL than the neutral contexts (non-interacting baseline and unstructured conversation), due to provoking more emotion and the need for interpersonal regulation.

### Expected Findings

In the present study, we observed both a simple in-phase PL pattern for IBI and a notably complex pattern. The predicted trajectories of IBI for the complex profile revealed temporally fine-grained nuances. Within 10-min conversations, we saw a relatively fast transition from in-phase synchronization to anti-phase synchronization, and then back to in-phase synchronization. Also, the partner’s oscillations eventually both damped, suggesting a regulatory process returning them toward their baseline after an initial perturbation. Although the exploratory nature of our study makes any interpretations speculative, such complex nuances may reflect a highly interactive coregulatory process in which the two partners were experiencing a range of emotions and exerting influence on each other in ways that were ultimately homeostatic.

It is also noteworthy that the simple profile was more common than the complex profile (i.e., 82 vs. 18% of all conversation contexts engaged by all couples). Such high prevalence of PL characterized by stable synchronization may reflect “business as usual” where partners were interacting in relatively unemotional ways not demanding of much self- or other- regulation. This is consistent with existing findings that relatively simple PL patterns can emerge when partners do not have to regulate each other’s behaviors and emotions (Parkinson, 2011). While this interpretation may account for the simple pattern emerging in the neutral contexts, in the more challenging contexts it is also possible that the simple pattern reflects a lack of engagement and hence a lack of coregulation. These interpretations gain some support from our findings that: (1) higher reports of love were associated with a higher probability of being in the complex profile, regardless of conversational context, and (2) higher reports of sexual satisfaction were associated with a higher probability of being in the complex profile during the body image conversation. Although the partners likely experienced and expressed intense feelings during the emotionally challenging body image conversations, partners experiencing security and interdependence may have been more effective at regulating each other’s emotions and behaviors, both actively and passively simply by providing a secure base for each other (Beckes and Coan, 2011; Timmons et al., 2015). In summary, as expected, the complex PL pattern was most likely to emerge for couples with high relationship quality during the challenging body image conversation, suggesting it may reflect coregulatory processes. In contrast, the simple PL pattern dominated for couples with lower relationship quality across contexts, suggesting a lack of engagement with each other. The simple PL pattern also dominated for all couples during the neutral baseline and unstructured conversations, suggesting that these contexts did not call for the more intense interpersonal engagement evoked by the body image conversation.

### Unexpected Findings

One unexpected finding from our study was that the health goal conversation was not associated with a higher probability of the complex profile. In fact, although more committed couples showed higher probability of being in the complex profile than low commitment couples when discussing health goals (as expected), all couples were more likely to be in the simple profile during this conversation, similar to the neutral baseline and unstructured conversations. A second unexpected finding was that there was no association between relationship length and the probability of being in the complex profile. On the one hand, there may be theoretical explanations for these null effects. For example, it may be that discussing health goals is not very challenging or engaging for same-sex male couples, especially if they are not very committed to each other. For relationship length, being together longer may reflect a more secure relationship and hence more capacity for coregulation, but it may also reflect a relationship that is hard to perturb and hence result in less coregulation. Such processes may have cancelled out in our study. And of course, as always with null results, they may simply reflect the low power of our relatively small sample. Future work with larger samples will be required to address this issue.

A third unexpected finding is that regardless of the conversational context, larger between-partner differences in their reports of habitual conflict were related to a higher probability of being in the complex profile. One possible interpretation of this finding is that those couples who disagreed on how much conflict they typically experience may have been struggling with the major relational task of constructing a shared reality and shared perceptions of their experiences (Acitelli et al., 1993; Wilson and Huston, 2013). Importantly, the only way to have a large discrepancy in reports of conflict is to have one of the partners reporting a fairly conflict-free relationship, suggesting that although the relationship may be facing a challenge, at least one of the partners is still optimistic about it. From this perspective, a large between-partner difference in the report and perception of conflict (i.e., an important and inevitable experience in couple relationships) may indicate a context in which at least the partner reporting less conflict was still enacting efforts to regulate emotion and behavior in the relationship, which in turn could be related to a high likelihood of complex PL patterns (Sbarra and Hazan, 2008; Holt-Lunstad et al., 2010; Coan and Sbarra, 2015). The unexpected nature of this finding precludes a strong interpretation, but this result suggests future work could systematically vary how much partners agree on key relationship aspects and test whether PL patterns vary as a result.

### Limitations and Future Directions

Several limitations of the present study are important to consider. First, we used a couple-centered approach to explore and describe PL patterns within the current sample. Given the relatively small sample size (i.e., 133 contexts completed by 34 couples) and the minority sample (e.g., same-sex male couples), the PL patterns identified cannot reflect the full range of complexity and diversity of PL patterns in interpersonal relationships. Instead, the two qualitatively different patterns identified in the current sample highlight the need for future studies in the field of PL that make use of methods capable of capturing the diversity of possible PL patterns.

Second, during the original data collection IBI was recorded from 72 same-sex male couples, but valid IBI data was only obtained from 34 of them (e.g., the sample used for the present analyses). The high missing data rate was primarily because of unexpected, random issues such as unstable signal transmission, excessive sweating, and movement artifacts. We investigated the potential bias introduced by the missing data with an attrition analysis and found no differences in relationship functioning indices, age, or relationship length between the couples who were included in the present study and the excluded couples. However, we acknowledge that the attrition rate is a unfortunate limitation in the present study.

Third, the measures of relationship functioning used in this study were assessed cross-sectionally before participation in the conversational contexts. Thus, we treated these indicators of relationship functioning as the antecedents for PL during each context. However, given the possible cyclical nature between couple relationship functioning and PL (Butler, 2017), it is inappropriate for us to speak about directionality. For example, it may be that relationship functioning impacted PL (as modeled), but it could also have been PL that influenced couple’s relationship functioning at a later time point. Future studies should be designed to assess the association between couple relationship functioning and PL in both directions.

Fourth, guided by social baseline theory and the reactive flexibility perspective, we argued that the emergence of complicated PL patterns may reflect efforts to regulate emotion during highly arousing contexts and among couples in well-functioning relationships, and may represent effective co-regulation. In contrast, we speculate that simple patterns may emerge in non-demanding situations or when partners are disengaged from each other. Such explanations are relatively speculative, however, and our exploratory approach can not tell us exactly why a specific pattern occurred. Evidence confirming or refuting these theoretical speculations will need to be gathered in future work using experimental and confirmatory methods. Nevertheless, our exploratory work points the way for such studies by demonstrating how to distinguish diverse PL patterns and suggesting factors that may be either a cause or a consequence of those patterns.

## Data Availability Statement

The data analyzed in this study is subject to the following licenses/restrictions: Data cannot be publicly available but are available upon contacting the fourth and fifth author. Requests to access these datasets should be directed to KA, kristin.august@rutgers.edu and CM, chmarkey@camden.rutgers.edu.

## Ethics Statement

The studies involving human participants were reviewed and approved by university of rugters. The patients/participants provided their written informed consent to participate in this study.

## Author Contributions

XL has finished the analyses and writing up the draft of the whole manuscript as the first author. AK and SB helped with revising the manuscript and searching for literature. KA and CM helped with cleaning up the data and revising the manuscript. EB, as the anchor author, has supervised XL during the whole process of data analyses, draft writing, and manuscript revising. All authors contributed to the article and approved the submitted version.

## Conflict of Interest

The authors declare that the research was conducted in the absence of any commercial or financial relationships that could be construed as a potential conflict of interest.
