# Corrigendum: Exploring Physiological Linkage in Same-Sex Male Couples

**DOI:** 10.3389/fpsyg.2021.720779

**Published:** 2021-07-13

**Authors:** Xiaomin Li, Ashley Kuelz, Savannah Boyd, Kristin August, Charlotte Markey, Emily Butler

**Affiliations:** ^1^Department of Family Studies and Human Development, The University of Arizona, Tucson, AZ, United States; ^2^Department of Psychology, The University of Arizona, Tucson, AZ, United States; ^3^Department of Psychology, Rutgers University, Camden, NJ, United States

**Keywords:** physiological linkage, relationship functioning, *rties* package, same-sex male couples, conversational context

In the original article, there was a mistake in the Figures as published. We accidentally deleted an important figure during the revision and resubmission process and did not catch this issue when proofreading. The correct Figure 1 appears below, which displays the predicted IBI trajectories for the two profiles we identified using person-centered analyses. The original Figures 1, 2, 3 and 4 should be regarded as Figures 2, 3, 4, and 5, respectively.

**Figure 1:**
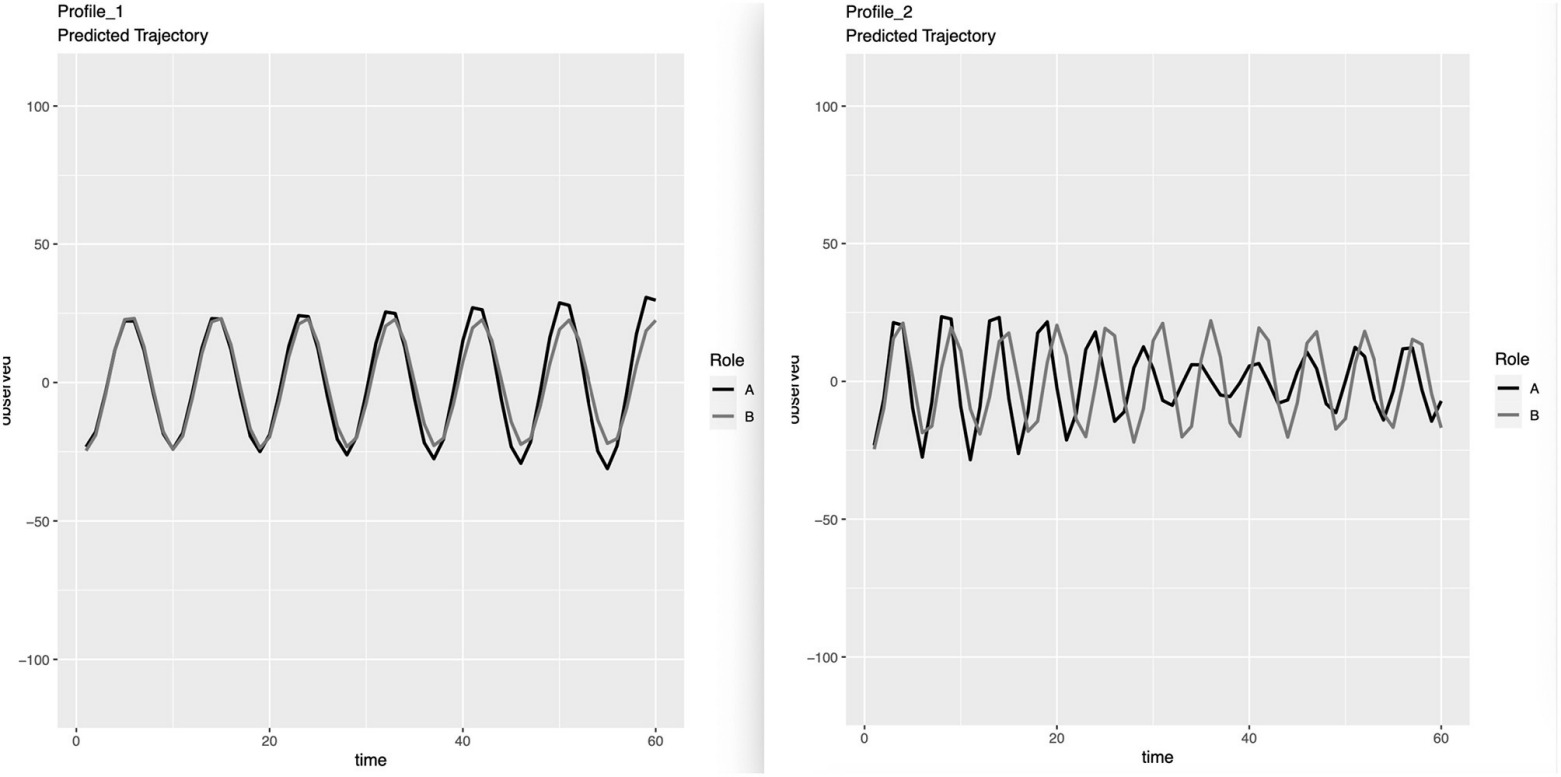
Estimated IBI trajectories for the two profiles. The first profile characterized 109 topic-couple combinations, while the second characterized 24. For indistinguishable dyads, the distinguishing variable A/B was randomly assigned and should not be interpreted. Predicted trajectories for Profile 1: The Simple Profile. Predicted trajectories for Profile 2: The Complex Profile.

The authors apologize for this error and state that this does not change the scientific conclusions of the article in any way.

