# Supplementary material for: Exploring Physiological Linkage in Same-Sex Male Couples
Source: Front Psychol. 2021 Jan 18;11:619255. doi: 10.3389/fpsyg.2020.619255 (PMC7848119; doi:10.3389/fpsyg.2020.619255)
Supplement: Supplementary file 1 [file Table_1.docx]

Supplementary Table 1. Level-1 specifications (e.g., fixed effects) for the multilevel models estimated in the present study. All models included a random couple intercept to account for nesting of topics in couples.

| **Panel A:** Models with different combinations of average love, between-partner difference in love, and topics as fixed predictors | | **Panel B:** Models with different combinations of average conflict, between-partner difference in conflict, and topics as fixed predictors | |
| --- | --- | --- | --- |
| *Model 1a* | Profile _topic i, dyad j_ = π_0j_ + π_10_ (Topic) + e_ij_ | *Model 1b* | Profile _topic i, dyad j_ = π_0j_ + π_10_ (Topic) + e_ij_ |
| *Model 2a* | Profile _topic i, dyad j_ = π_0j_ + π_20_ (Average love) + π_30_ (Between-partner difference in love) + e_ij_ | *Model 2b* | Profile _topic i, dyad j_ = π_0j_ + π_20_ (Average conflict) + π_30_ (Between-partner difference in conflict) + e_ij_ |
| *Model 3a* | Profile _topic i, dyad j_ = π_0j_ + π_10_ (Topic) + π_20_ (Average love) + π_30_ (Between-partner difference in love) + π_40_ (Topic) × (Average love) + π_50_ (Topic) × (Between-partner difference in love) + e_ij_ | *Model 3b* | Profile _topic i, dyad j_ = π_0j_ + π_10_ (Topic) + π_20_ (Average conflict) + π_30_ (Between-partner difference in conflict) + π_40_ (Topic) × (Average conflict)  + π_40_ (Topic) × (Between-partner difference in conflict)  + e_ij_ |
|  |  | *Simplified*  *Model 3b* | Profile _topic i, dyad j_ = π_0j_ + π_10_ (Topic) + π_20_ (Average conflict) + π_30_ (Between-partner difference in conflict) + π_40_ (Topic) × (Average conflict)  + e_ij_ |
| **Panel C:** Models with different combinations of average sexual satisfaction, between-partner difference in sexual satisfaction, and topics as fixed predictors | | **Panel D:** Models with different combinations of average commitment, between-partner difference in commitment, and topics as fixed predictors | |
| *Model 1c* | Profile _topic i, dyad j_ = π_0j_ + π_10_ (Topic) + e_ij_ | *Model 1d* | Profile _topic i, dyad j_ = π_0j_ + π_10_ (Topic) + e_ij_ |
| *Model 2c* | Profile _topic i, dyad j_ = π_0j_ + π_20_ (Average sexual satisfaction) + π_30_ (Between-partner difference in sexual satisfaction) + e_ij_ | *Model 2d* | Profile _topic i, dyad j_ = π_0j_ + π_20_ (Average commitment) + π_30_ (Between-partner difference in commitment) + e_ij_ |
| *Model 3c* | Profile _topic i, dyad j_ = π_0j_ + π_10_ (Topic) + π_20_ (Average sexual satisfaction)  + π_30_ (Between-partner difference in sexual satisfaction)  + π_40_ (Topic) × (Average sexual satisfaction)  + π_50_ (Topic) × (Between-partner difference in sexual satisfaction)  + e_ij_ | *Model 3d* | Profile _topic i, dyad j_ = π_0j_ + π_10_ (Topic) +  π_20_ (Average commitment)  + π_30_ (Between-partner difference in commitment)  + π_40_ (Topic) × (Average commitment)  + π_50_ (Topic) × (Between-partner difference in  commitment)  + e_ij_ |
| *Simplified*  *Model 3c* | Profile _topic i, dyad j_ = π_0j_ + π_10_ (Topic) + π_20_ (Average sexual satisfaction)  + π_30_ (Between-partner difference in sexual satisfaction)  + π_40_ (Topic) × (Average sexual satisfaction)  + e_ij_ | *Model 3d* | Profile _topic i, dyad j_ = π_0j_ + π_10_ (Topic) +  π_20_ (Average commitment)  + π_30_ (Between-partner difference in commitment)  + π_40_ (Topic) × (Average commitment)  + e_ij_ |
| **Panel E:** Models with different combinations of relationship length and topics as fixed predictors | |  |  |
| *Model 1e* | Profile _topic i, dyad j_ = π_0j_ + π_10_ (Topic) + π_20_ (Relationship length) + e_ij_ |  |  |
| *Model 2e* | Profile _topic i, dyad j_ = π_0j_ + π_10_ (Topic) + π_20_ (Relationship length) + π_30_ (Average love) × (Relationship length) + e_ij_ |  |  |

*Note.* In Panel B, (Topic) × (Between-partner difference in conflict) did not have notable effects, so we trimmed it and generated simplified Model 3b. In Panel C, (Topic) × (Between-partner difference in sexual satisfaction) did not have notable effects, so we trimmed it and generated simplified Model 3c. Similarly, in Panel D, (Topic) × (Between-partner difference in commitment) did not have notable effects, so we trimmed it and generated simplified Model 3d.
