# Supplementary material for: Exploring Physiological Linkage in Same-Sex Male Couples
Source: Front Psychol. 2021 Jan 18;11:619255. doi: 10.3389/fpsyg.2020.619255 (PMC7848119; doi:10.3389/fpsyg.2020.619255)
Supplement: Supplementary file 2 [file Table_2.docx]

Supplementary Table 2. Summary of R^2^ and cross-validation of models within each panel. The model with smallest eldp LOO is displayed in the first row.

| **Panel A:** Models with different combinations of average love, between-partner difference in love, and topics as fixed predictors | | | | | **Panel B:** Models with different combinations of average conflict, between-partner difference in conflict, and topics as fixed predictors | | |
| --- | --- | --- | --- | --- | --- | --- | --- |
|  | | *R^2^*  with 95% HDI | | Difference in elpd LOO from 1^st^ Row (SE of difference) |  | *R^2^*  with 95% HDI | Difference in elpd LOO from 1^st^ Row (SE of difference) |
| ***Model 2a*** | | | **.20**  **[.04, .35]** | **--** | ***Model 2b*** | **.20**  **[.06, .35]** | **--** |
| *Model 1a* | | | .25  [.07, 41] | -0.2 (3.9)  Difference is 0.05 SE | *Model 1b* | .25  [.07, .41] | -2.1 (4.3)  Difference is 0.49 SE |
| *Model 3a* | | | .35  [.18, .47] | -14.1 (7.9)  Difference is 1.9 SEs | *Simplified Model 3b* | .37  [.21, .49] | -5.1 (5.7)  Difference is 0.9 SE |
|  | | |  |  | *Model 3b* | .40  [.25, .52] | -12.2 (7.1)  Difference is 1.7 SEs |
| **Panel C:** Models with different combinations of average sexual satisfaction, between-partner difference in sexual satisfaction, and topics as fixed predictors | | | | | **Panel D:** Models with different combinations of average commitment, between-partner difference in commitment, and topics as fixed predictors | | |
|  | | | *R^2^*  with 95% HDI | Difference in elpd LOO from 1^st^ Row (SE of difference) |  | *R^2^*  with 95% HDI | Difference in elpd LOO from 1^st^ Row (SE of difference) |
| *Model 1c* | | | .26  [.08, .42] | -- | *Model 2d* | .18  [.03, .34] | -- |
| *Model 2c* | | | .20  [.04, .35] | -0.3 (3.6)  Difference is 0.1 SE | *Model 1d* | .26  [.07, .41] | -0.5 (4.0)  Difference is 0.1 SE |
| ***Simplified Model 3c*** | | | **.37**  **[.21, .49]** | **-5.2 (4.1)**  **Difference is 1.3 SEs** | ***Simplified***  ***Model 3d*** | **.35**  **[.19, .48]** | **-2.4 (5.1)**  **Difference is 0.5 SE** |
| *Model 3c* | | | .41  [.27, 53] | -17.1 (7.9)  Difference is 2.2 SEs | *Model 3d* | .39  [.24, .51] | -7.7 (6.5)  Difference is 1.2 SEs |
| **Panel E:** Models with different combinations of relationship length and topics as fixed predictors | | | | |  |  |  |
|  | *R^2^* with 95% HDI | | | Difference in elpd LOO from 1^st^ Row (SE of difference) |  |  |  |
| ***Model 1e*** | **.26**  **[.08. .41]** | | | **--** |  |  |  |
| *Model 2e* | .29  [.12, .43] | | | -4.6 (3.3)  Difference is 1.4 SEs |  |  |  |

*Note.* Optimal models are in bold.

HDI = High density interval, elpd LOO = expected log predictive density for leave-one-out cross-validation, and SE = standard error.
